# Prognostic Impact and Postoperative Management Following Poor Pathological Response to Perioperative FLOT in Resectable Gastric and GEJ Adenocarcinoma: A Systematic Review and Meta-Analysis

**DOI:** 10.3390/jcm15062367

**Published:** 2026-03-20

**Authors:** Ismaell Massalha, Reem Zabit, Samer Hussany, Adham Hijab, Wael Hozaeel, Israel Sandler, Jamal Zidan, Ory Wiesel, Ravit Geva

**Affiliations:** 1 Department of Oncology, Ziv Medical Center, Safed 13100, Israeljamalz@ziv.gov.il (J.Z.); 2 Department of Medical Bioinformatics, Ben-Gurion University of the Negev, Be’er Sheva 8410501, Israel; 3 Department of Pediatrics, Ziv Medical Center, Safed 13100, Israel; 4 Oncology Institute, Soroka University Medical Center, Be’er Sheva 8410101, Israel; 5 Department of Nuclear Medicine, Ziv Medical Center, Safed 13100, Israel; 6 Baruch Padeh-Tzafon Medical Center, Poriya 1528001, Israel; orywiesel@gmail.com; 7 Department of Oncology, Tel Aviv Sourasky Medical Center, Tel Aviv 6423906, Israel; ravitg@tlvmc.gov.il

**Keywords:** gastric cancer, gastroesophageal junction, perioperative chemotherapy, FLOT, tumor regression grade, pathological response, systematic review, meta-analysis, prognosis, adjuvant chemotherapy

## Abstract

**Background:** Pathological tumor regression is a key prognostic marker in resectable gastric and gastroesophageal junction (GEJ) adenocarcinoma. Perioperative FLOT (fluorouracil, leucovorin, oxaliplatin, docetaxel) established the modern treatment backbone, and the recent MATTERHORN trial further intensified this paradigm with the addition of durvalumab. However, a substantial proportion of patients fail to achieve major pathological regression, and the prognostic magnitude of poor response in the FLOT era has not been systematically quantified. **Methods:** We performed a PRISMA 2020–compliant, PROSPERO registered (CRD420251150054) systematic review and meta-analysis. PubMed, Web of Science, Scopus, and Cochrane were searched through September 2025 for studies including patients with resectable gastric/GEJ adenocarcinoma treated with perioperative FLOT followed by curative surgery. Associations between poor pathological response and survival were quantified. Exploratory analyses evaluated completion of postoperative FLOT versus incomplete therapy. Random effects models were applied to pooled hazard ratios (HRs). **Results:** Twelve studies comprising 4201 resected patients were included in this systematic review; 1817 (43.2%) were classified as poor responders. Poor pathological response was strongly associated with inferior outcomes: pooled HR for overall survival (OS) was 2.73 (95% CI 2.18–3.43; $I^{2}=0.0\%$) and pooled HR for disease-free/recurrence-free survival (DFS/RFS) was 2.68 (95% CI 2.14–3.34; $I^{2}=51.2\%$). Effect direction was consistent across Becker, Mandard, and CAP grading systems. Exploratory analysis of three observational cohorts found an association between completion of postoperative FLOT and improved survival (HR 0.49, 95% CI 0.31–0.79); however, this comparison is inherently confounded by postoperative fitness and treatment selection and should not inform clinical decision-making. **Conclusions:** Poor pathological response after perioperative FLOT identifies a large, high risk subgroup with approximately threefold inferior survival despite R0 resection. These findings establish contemporary prognostic benchmarks and underscore the absence of prospective evidence guiding postoperative management in poor responders.

## 1. Introduction

Perioperative chemotherapy with fluorouracil, leucovorin, oxaliplatin, and docetaxel (FLOT) established a modern therapeutic backbone for resectable gastric and gastroesophageal junction (GEJ) adenocarcinoma following the pivotal FLOT4 trial, which demonstrated superior survival and higher pathological response rates compared with anthracycline based regimens [1,2]. Subsequent real-world studies confirmed its feasibility and broad adoption in clinical practice [3,4,5]. Although perioperative strategies are now evolving toward chemoimmunotherapy combinations, FLOT remains the cytotoxic platform upon which contemporary regimens are constructed. Consequently, pathological response after preoperative therapy continues to represent a central biological and prognostic readout in this disease.

The phase III MATTERHORN trial recently demonstrated improved event-free survival and pathological complete response rates with the addition of durvalumab to perioperative FLOT [6]. Importantly, subgroup analyses did not demonstrate a statistically significant interaction according to PD-L1 (Programmed Death-Ligand 1) expression or microsatellite instability (MSI) status, although these subgroups were relatively small. Despite therapeutic intensification, a substantial proportion of patients fail to achieve major pathological regression, and the clinical implications of residual viable disease remain critical.

Tumor regression grading (TRG) systems, including Becker, Mandard, and College of American Pathologists (CAP) classifications, quantify residual tumor burden after neoadjuvant therapy. Multiple cohorts have associated poor pathological response (commonly defined as Becker 2–3 or Mandard ≥ 3) with inferior survival despite R0 resection [7,8,9,10]. Real-world data suggest that approximately 40–50% of resected patients fall within poor response categories [11,12]. However, the magnitude of prognostic risk in the FLOT-specific era has not been systematically synthesized.

Postoperative management of poor responders remains undefined. The FLOT protocol includes four postoperative cycles identical to the preoperative regimen; however, completion rates vary and survival benefit from continuation, modification, or escalation has not been prospectively established [13]. Retrospective studies exploring postoperative chemoradiotherapy, treatment intensification, or immunotherapy continuation are heterogeneous and limited by selection bias [14,15,16]. Current clinical guidelines acknowledge poor response as an adverse factor but do not provide TRG guided postoperative algorithms [17]. Variability in TRG reporting and inconsistent postoperative stratification further impede evidence synthesis [18,19,20].

Conceptually, analogous paradigms in other solid tumors suggest that biologically defined high-risk residual disease may represent a rational target for postoperative intervention. In other malignancies, residual disease phenotypes after multimodal therapy have informed treatment intensification strategies; whether similar response-adapted approaches will be relevant in resectable gastric or GEJ adenocarcinoma remains unproven and requires disease-specific prospective validation.

We therefore conducted a systematic review and meta-analysis to (i) quantify the prognostic magnitude of poor pathological response following perioperative FLOT and (ii) evaluate available evidence regarding postoperative management within this subgroup. Establishing grading system-independent risk estimates in the chemotherapy-defined FLOT era provides a necessary benchmark for interpreting outcomes and for designing response-adapted strategies within evolving perioperative treatment paradigms.

## 2. Methods

### 2.1. Reporting Standard and Protocol Registration

This systematic review and meta-analysis was conducted and reported in accordance with the Preferred Reporting Items for Systematic Reviews and Meta Analyses (PRISMA) 2020 statement [21]. The protocol was prospectively registered in the International Prospective Register of Systematic Reviews (PROSPERO; registration number CRD420251150054) prior to data extraction [22]. No major deviations from the registered protocol occurred.

### 2.2. Clinical Question and Eligibility Framework

The primary clinical question was: *In adults with resectable gastric or gastroesophageal junction (GEJ) adenocarcinoma treated with perioperative or neoadjuvant FLOT chemotherapy who demonstrate poor pathological response, what are the associated survival outcomes, and what evidence exists regarding postoperative management strategies within this subgroup?*

Eligibility criteria were defined using a PICO framework:

- **Population:** Adults (≥18 years) with resectable gastric or GEJ adenocarcinoma (including Siewert type II–III).
- **Exposure:** Perioperative or neoadjuvant FLOT-based chemotherapy followed by curative intent surgery.
- **Comparisons:** (i) Poor pathological responders versus responders and (ii) exploratory comparisons of postoperative strategies within poor responders.
- **Outcomes:** Overall survival (OS) as the primary endpoint; disease-free survival (DFS), recurrence-free survival (RFS), progression-free survival (PFS), recurrence patterns, postoperative outcomes, and reported clinical or molecular correlates.

### 2.3. Information Sources and Search Strategy

A systematic search of the literature was conducted in PubMed/MEDLINE, Web of Science, Scopus, and the Cochrane Library from database inception through 17 September 2025. No language or publication date restrictions were applied. Reference lists of included studies and relevant reviews were manually screened to identify additional eligible reports.

The complete PubMed/MEDLINE search strategy is provided below to ensure reproducibility:

(“gastric cancer”[Title/Abstract] OR “gastroesophageal junction cancer”[Title/ Abstract] OR “stomach neoplasms”[MeSH Terms] OR “gastric adenocarcinoma”[Title/Abstract] OR “GEJ cancer”[Title/Abstract] OR “oesophagogastric cancer”[Title/Abstract]) AND (“FLOT”[Title/Abstract] OR (“docetaxel”[Title/ Abstract] OR “oxaliplatin”[Title/Abstract] OR “leucovorin”[Title/Abstract] OR “5-fluorouracil”[Title/Abstract]) OR “perioperative chemotherapy”[Title/ Abstract] OR “neoadjuvant chemotherapy”[Title/Abstract] OR “adjuvant chemotherapy”[Title/Abstract] OR “triplet chemotherapy”[Title/Abstract]) AND (“tumor regression grade”[All Fields] OR “TRG”[All Fields] OR “pathologic response”[All Fields] OR “histopathologic response”[All Fields] OR “responder”[All Fields] OR “non-responder”[All Fields] OR “partial response”[All Fields] OR “pCR”[All Fields] OR “treatment response”[All Fields]) AND (“R0 resection”[All Fields] OR “curative surgery”[All Fields] OR “radical surgery”[All Fields] OR “overall survival”[All Fields] OR “OS”[All Fields] OR “progression free survival”[All Fields] OR “PFS”[All Fields] OR “disease free survival”[All Fields])

Full search strategies for Web of Science, Scopus, and the Cochrane Library are provided in the Supplementary Materials.

Across all databases, 1528 records were identified. After the removal of 803 duplicates, 725 unique records underwent screening. The search strategy was intentionally broad to maximize sensitivity; studies not reporting FLOT-specific outcomes were excluded at full-text screening if not already identified during title and abstract review.

### 2.4. Eligibility Criteria

Studies were eligible if they met the following criteria:

1. Included adults with resectable gastric or GEJ adenocarcinoma;
2. Reported treatment with perioperative or neoadjuvant FLOT;
3. Included patients undergoing curative intent resection;
4. Reported pathological response using a recognized tumor regression grading (TRG) system (e.g., Becker, Mandard, CAP) or a clearly defined surrogate permitting identification of poor responders;
5. Reported survival or recurrence outcomes.

Randomized controlled trials and prospective or retrospective cohort studies were eligible. We excluded case reports, narrative reviews, editorials, conference abstracts without extractable data, studies evaluating non-FLOT regimens without separable FLOT-specific results, and studies lacking extractable pathological response or relevant oncologic outcomes.

### 2.5. Study Selection

Study selection was conducted in two stages: title/abstract screening followed by full-text review. Screening and eligibility assessment were performed independently by two reviewers. Disagreements were resolved by discussion and consensus.

Given the limited number of contemporary FLOT-specific cohorts, studies meeting predefined eligibility criteria were retained despite heterogeneity in TRG definitions or reporting. Heterogeneity was addressed analytically through prespecified subgroup and sensitivity analyses.

### 2.6. Data Extraction

Data extraction was performed independently by two reviewers using a predefined template. Extracted variables included the following:

- Study characteristics (author, year, country, design, sample size, median follow up);
- Treatment details (FLOT protocol, surgical approach, R0 resection rate, postoperative therapy completion);
- Pathological response definitions and grading systems;
- Survival outcomes (OS, DFS/RFS/PFS) and corresponding hazard ratios (HRs) with 95% confidence intervals;
- Recurrence patterns and reported biomarkers (e.g., HER2, PD-L1, MSI, Claudin18.2), when available.

Adjusted HRs were preferentially extracted to mitigate confounding. For studies not directly reporting HRs, log(HR) and corresponding standard errors were reconstructed from published summary statistics using established methods described by Parmar et al. and Tierney et al. Sensitivity analyses excluding reconstructed estimates were performed to assess robustness.

### 2.7. Definition and Harmonization of Pathological Response

Because different TRG systems were used across studies, we prespecified a harmonized definition of poor pathological response. Poor response was defined as follows:

- Becker TRG 2–3;
- Mandard TRG 3 or more;
- CAP TRG indicating minimal or no tumor regression.

These systems differ in their histological thresholds and are not strictly interchangeable: Becker TRG 2–3 reflects less than 50% regression, while Mandard TRG 3–5 encompasses a broader range of residual tumor burden. The harmonized category therefore does not represent a perfectly identical biological population across studies, and the potential for misclassification should be acknowledged when interpreting pooled estimates. The harmonization was intended to pragmatically capture the adverse end of the response spectrum, defined by substantial residual viable tumor after preoperative therapy. Pooled estimates should be read as summarizing prognosis among patients with limited pathological regression rather than as an effect size for a strictly uniform response phenotype. Subgroup analyses stratified by TRG system were conducted to evaluate consistency of effect estimates across frameworks.

### 2.8. Risk of Bias Assessment

Risk of bias for observational studies was independently assessed by two reviewers using the Newcastle–Ottawa Scale (NOS), evaluating cohort selection, comparability, and outcome assessment. Domain-level judgments and total star scores are reported in the Supplementary Materials to contextualize evidence strength. NOS results were not used as exclusion criteria.

### 2.9. Statistical Analysis

Two prespecified analyses were conducted:

1. Prognostic association between poor pathological response and survival outcomes.
2. Exploratory comparisons of postoperative strategies among poor responders, specifically completion versus non-completion of planned postoperative FLOT.

Meta-analysis was performed when at least two studies reported compatible HRs for a given endpoint. Otherwise, findings were summarized narratively.

All prognostic analyses were restricted to resected patients, reflecting the post-resection clinical decision making context. Pooled estimates therefore represent post-resection prognostic associations rather than population-level treatment effects.

Random effects models with restricted maximum likelihood (REML) estimation were used to account for anticipated clinical and methodological heterogeneity. Between-study heterogeneity was quantified using $I^{2}$ and $\tau^{2}$.

Prespecified subgroup analyses were conducted according to TRG classification system (Becker, Mandard, CAP). Meta regression analyses explored study-level moderators, including covariate adjustment status, proportion of patients not undergoing surgery, and reported R0 resection rate.

Publication bias was evaluated using funnel plots and Egger’s regression test when ≥5 studies were available for a given endpoint. Trim and fill analyses were performed as sensitivity analyses. Additional sensitivity analyses included leave-one-out procedures and the exclusion of statistical outliers.

All analyses were performed using R (R Foundation for Statistical Computing, Vienna, Austria) with the metafor and meta packages.

## 3. Results

### 3.1. Study Selection

**Figure 1:**
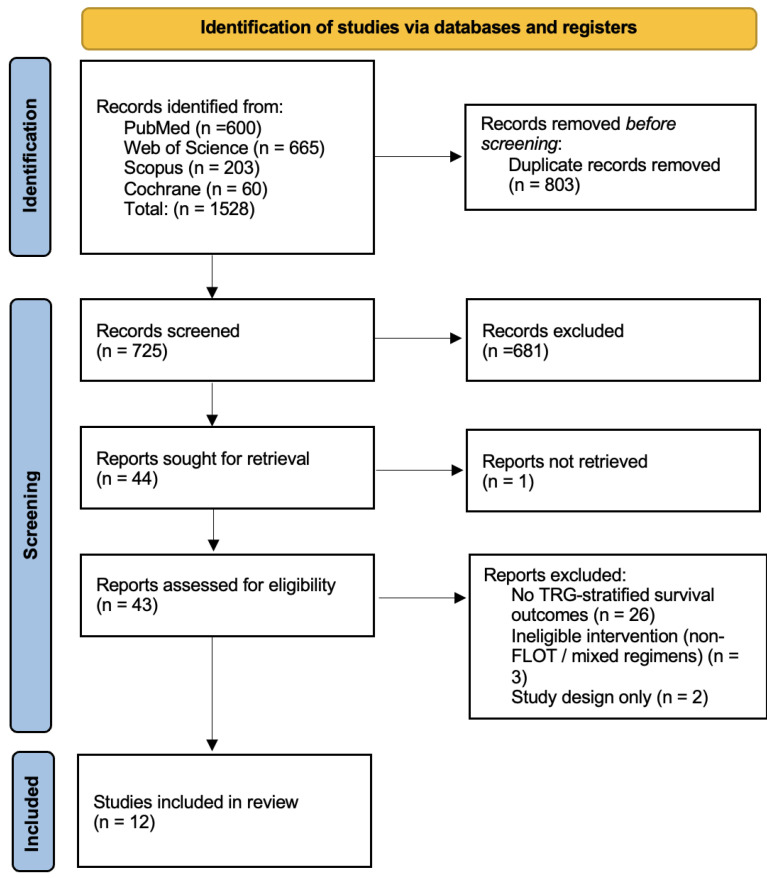
PRISMA 2020 flow diagram.

The PRISMA flow diagram is shown in Figure 1. The most common reason for exclusion at full-text review was the absence of survival outcomes reported stratified by pathological response category.

### 3.2. Study Characteristics and Prevalence of Poor Pathological Response

**Figure 2:**
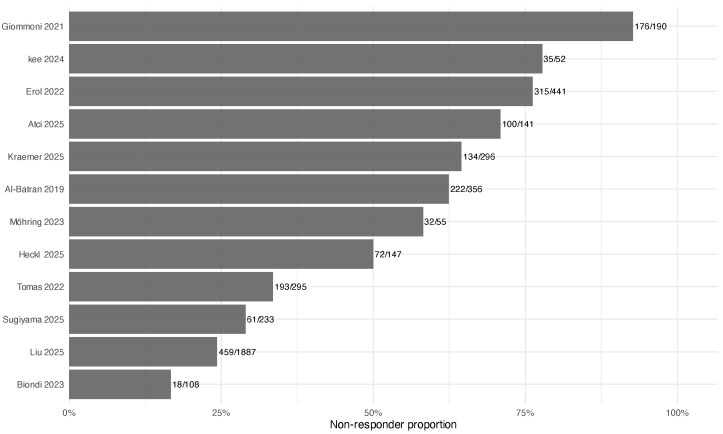
Proportion of poor pathological responders across included studies: Al-Batran 2019 [1], Giommoni 2021 [3], Erol 2022 [4], Tomás 2022 [23], Biondi 2023 [25], Möhring 2023 [5], Kee 2024 [26], Atci 2025 [27], Heckl 2025 [24], Kraemer 2025 [13], Liu 2025 [28], Sugiyama 2025 [29].

Twelve studies met the inclusion criteria, yielding data for 4201 resected patients treated with FLOT-based perioperative regimens (Table 1). Overall, 1817 patients (43.2%) were classified as poor pathological responders according to study-specific TRG definitions. Reported median follow-up ranged from 12 to 45 months; follow-up was not reported in two studies (Tomas 2022 [23], Heckl 2025 [24]). The proportion of poor responders across studies is shown in Figure 2.

The proportion of patients classified as poor responders varied substantially across cohorts, partly reflecting non-equivalent underlying definitions. Giommoni et al. grouped all non-pCR patients into the poor-response category, yielding a prevalence of 92.7%, whereas Biondi et al. restricted classification to Mandard TRG 5, representing only 16.7% of that cohort. These differing thresholds capture different portions of the residual-disease spectrum and contribute to the between-study variation in poor-responder prevalence observed in Figure 2.

### 3.3. Oncologic Outcomes

#### 3.3.1. Prognostic Association: Poor Responders Versus Responders

**Figure 3:**
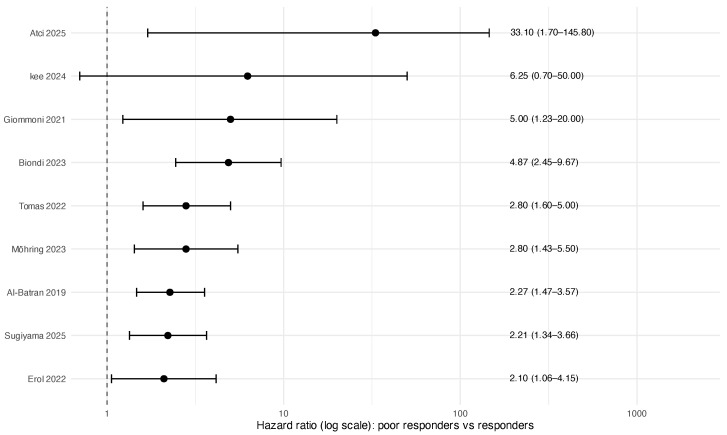
Forest plot of prognostic OS hazard ratios (poor responders vs. responders): Al-Batran 2019 [1], Erol 2022 [4], Tomás 2022 [23], Biondi 2023 [25], Möhring 2023 [5], Giommoni 2021 [3], Kee 2024 [26], Atci 2025 [27], Sugiyama 2025 [29].

**Figure 4:**
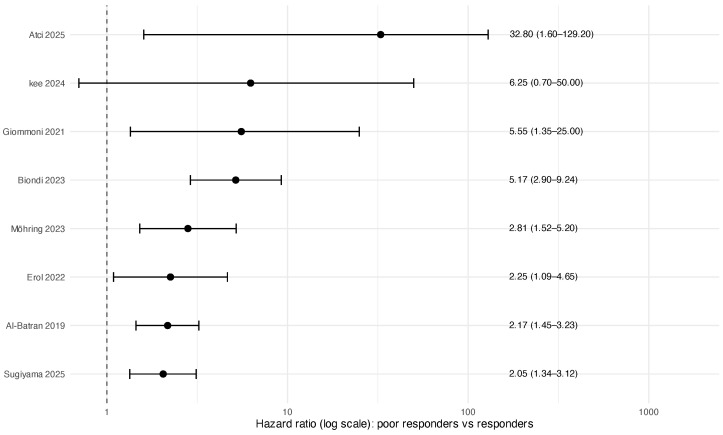
Forest plot of prognostic DFS/RFS hazard ratios (poor responders vs. responders): Al-Batran 2019 [1], Erol 2022 [4], Biondi 2023 [25], Möhring 2023 [5], Giommoni 2021 [3], Kee 2024 [26], Atci 2025 [27], Sugiyama 2025 [29].

Nine studies contributed overall survival (OS) estimates and eight contributed disease-control endpoints (DFS/RFS, pooled as reported). Poor pathological response was consistently associated with inferior outcomes. The pooled HR for OS was 2.73 (95% CI 2.18–3.43; $I^{2}=0.0\%$), and the pooled HR for DFS/RFS was 2.68 (95% CI 2.14–3.34; $I^{2}=51.2\%$) (Table 2; Figure 3 and Figure 4). As prespecified, analyses were restricted to resected patients; pooled estimates therefore represent post-resection prognostic associations rather than treatment effects in the intention-to-treat population.

#### 3.3.2. Consistency Across TRG Systems and Robustness Analyses

**Figure 5:**
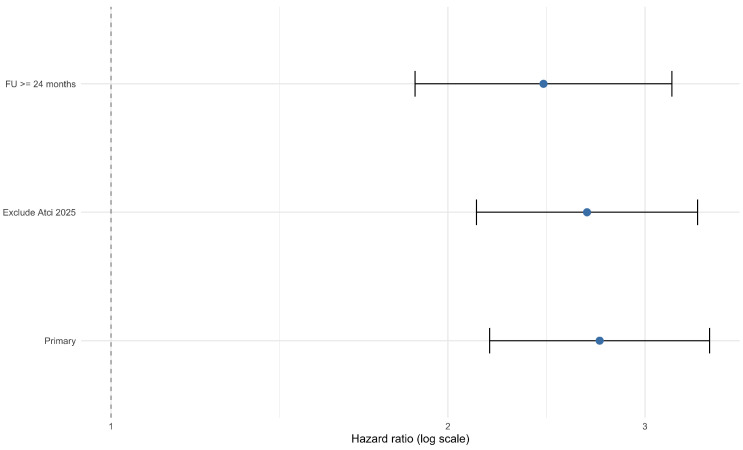
Sensitivity analyses of pooled OS hazard ratios [27].

Subgroup analyses by TRG system demonstrated a consistent direction of effect across Becker, Mandard, and CAP classifications (Table 3); however, precision varied substantially, particularly for CAP-based estimates, due to small study numbers and wide confidence intervals. Sensitivity analyses (leave-one-out and prespecified restrictions) did not materially change the pooled OS estimate (Table 4; Figure 5).

One cohort (Atci 2025 [27]) reported an unusually large hazard ratio (HR 33.10; 95% CI 1.70–645.80) with a confidence interval spanning two orders of magnitude. This estimate derives from univariable analysis in a relatively small cohort and likely reflects statistical instability due to a limited number of events rather than a true biological effect.

Leave-one-out analysis confirmed that exclusion of this study did not materially alter the pooled estimate. Restricting the analysis to cohorts with a median follow-up of at least 24 months attenuated the pooled OS hazard ratio from 2.73 to 2.43, which may partly reflect more complete event capture in longer-followed cohorts rather than a weaker underlying prognostic association.

**Figure 6:**
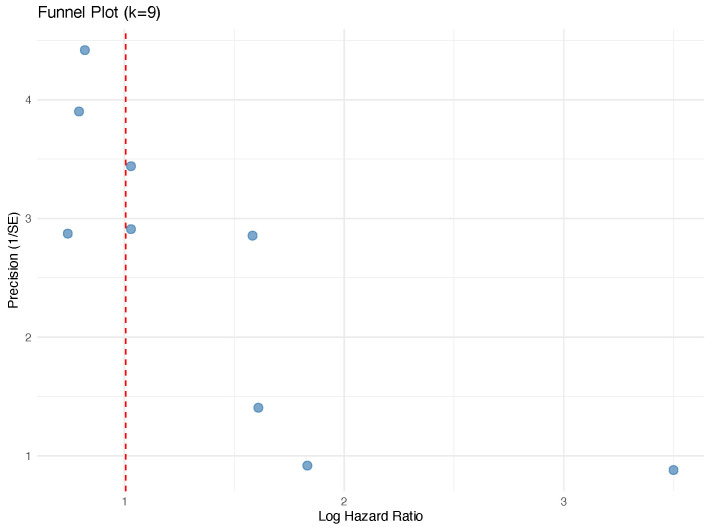
Funnel plot for assessment of publication bias (OS).

Meta-regression did not identify significant moderators of OS effect size (all p>0.4; Table 5), recognizing limited power. Funnel plot inspection and Egger’s regression did not suggest small-study effects for OS, and trim-and-fill did not impute missing studies (Figure 6).

#### 3.3.3. Postoperative Strategies Within Poor Pathological Responders (Exploratory)

**Figure 7:**
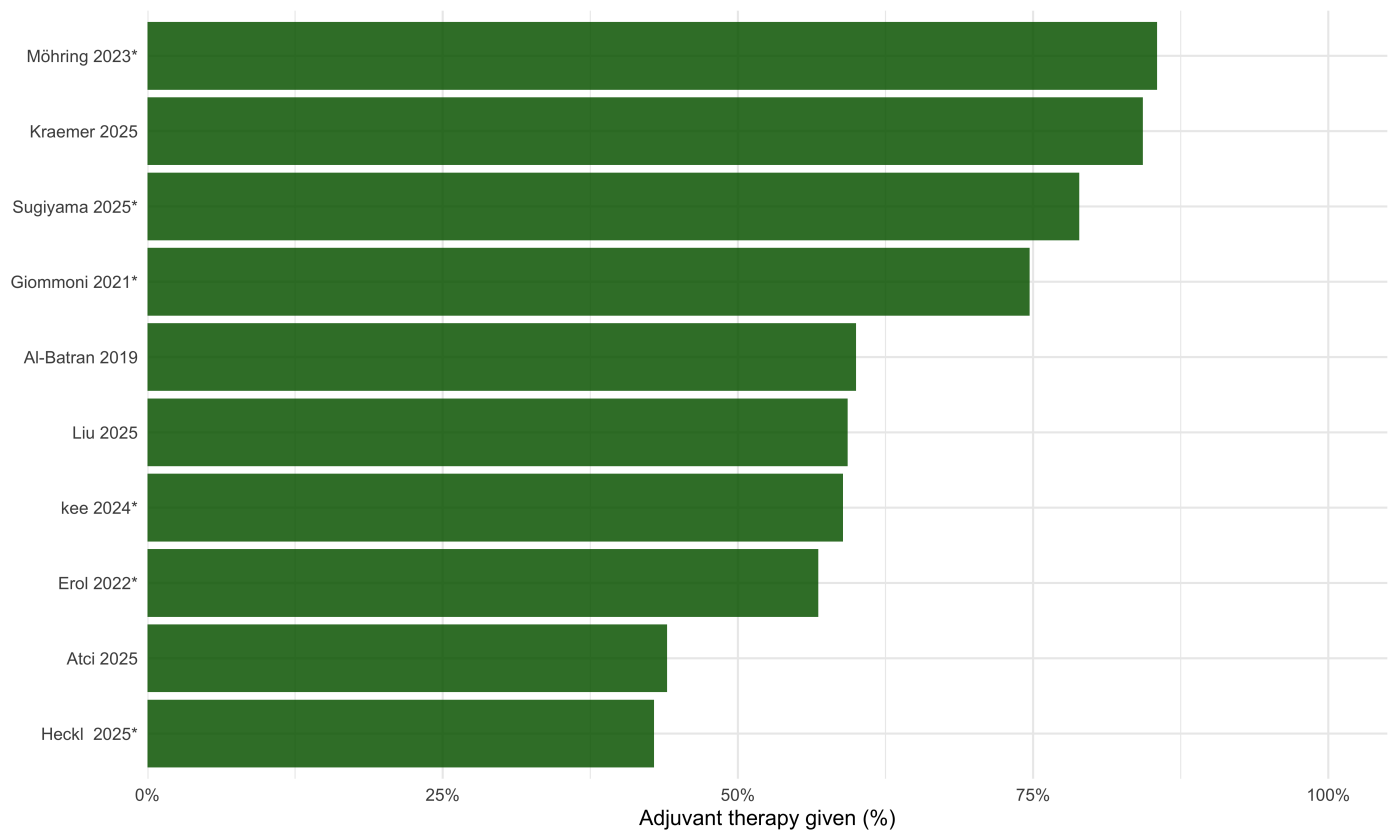
Postoperative therapy administration among poor pathological responders: Al-Batran 2019 [1], Giommoni 2021 [3], Erol 2022 [4], Kee 2024 [26], Atci 2025 [27], Heckl 2025 [24], Kraemer 2025 [13], Liu 2025 [28], Sugiyama 2025 [29], Möhring 2023 [5]. * are derived from overall cohorts rather than poor responder–specific reporting.

**Figure 8:**
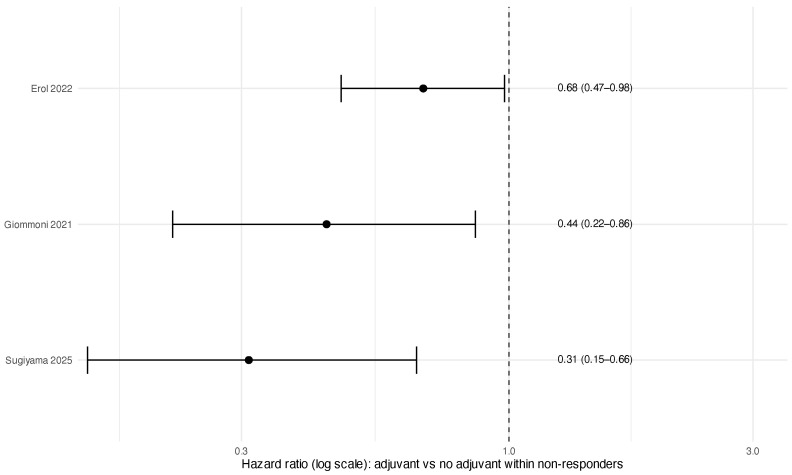
Forest plot of completion of postoperative FLOT cycles versus no or incomplete postoperative therapy within poor pathological responders (exploratory): Erol 2022 [4], Giommoni 2021 [3], Sugiyama 2025 [29].

Reporting of postoperative strategies within poor responders was inconsistent and comparisons were observational. Rates of adjuvant therapy administration across included studies are shown in Figure 7. Three studies provided extractable HRs comparing the completion of planned postoperative FLOT cycles to no or incomplete postoperative therapy [3,4,29]. Pooled analysis suggested an association with improved survival (HR 0.49, 95% CI 0.31–0.79; $I^{2}=50.1\%$) (Table 6; Figure 8). Given potential confounding by postoperative fitness, complications, and clinician selection, this finding should be interpreted as hypothesis-generating rather than as evidence of a causal benefit.

### 3.4. Risk of Bias and Reporting of Adjusted Covariates and Biomarkers

The risk of bias assessment using the Newcastle–Ottawa Scale is summarized in Supplementary Table S1. Selection and outcome ascertainment were generally adequate across cohorts; the most frequent limitation was reduced comparability due to heterogeneous or incomplete covariate adjustment.

The adjusted covariates included in the multivariable models are summarized in Table 7. Reporting of molecular biomarkers (including HER2, PD-L1, MSI, and Claudin18.2) was sparse and inconsistent across cohorts and was rarely stratified by pathological response category; a study-level summary is provided in Supplementary Table S7. The limited completeness of these data precluded quantitative synthesis.

Descriptive distributions of tumor biology and post-treatment pathological staging among poor responders are shown in Supplementary Figures S6 and S7. Across studies reporting these variables, poor responders were characterized by high rates of ypT3–4 and ypN+ disease, frequent lymphovascular and perineural invasion, and a predominance of intestinal-type histology.

## 4. Discussion

This systematic review and meta-analysis synthesizes contemporary evidence on patients with poor pathological response following perioperative FLOT, a subgroup comprising approximately two fifths (43.2%) of resected patients across included cohorts. To our knowledge, this represents the first quantitative synthesis defining the prognostic magnitude of poor pathological regression specifically within the modern FLOT era. By pooling time-to-event estimates from recent studies, the present analysis provides a clinically relevant benchmark for survival expectations in this high-risk population and clarifies the current evidentiary limits surrounding postoperative management.

### 4.1. Key Findings

Three principal observations merit emphasis.

Firstly, poor pathological response after perioperative FLOT is a strong and reproducible adverse prognostic marker among resected patients. Across heterogeneous TRG systems, poor responders experienced nearly threefold worse overall survival, with minimal statistical heterogeneity in the primary model. Although CAP-based estimates demonstrated wider confidence intervals, likely reflecting smaller sample sizes and broader categorical definitions [8,20], our subgroup analyses validate pooling these systems. Despite distinct histological benchmarks across the Becker, Mandard, and CAP scales, the direction and magnitude of the survival hazard remained highly consistent across all three (Table 3). This confirms that they effectively capture the same extreme biological phenotype of minimal-to-no treatment response.

Secondly, the therapeutically pivotal question of how to manage poor responders postoperatively remains unresolved. The FLOT protocol mandates four postoperative cycles identical to the preoperative regimen; however, completion rates vary in real-world practice, and TRG-stratified postoperative outcome reporting is inconsistent. Exploratory pooling of three observational cohorts [13,28,29] suggested an association between completion of postoperative FLOT cycles and improved survival among poor responders. This association is inherently confounded by indication: patients who complete postoperative therapy typically have better performance status, fewer surgical complications, and superior recovery trajectories. The observed signal should not be read as evidence of the efficacy of continuing an unresponsive systemic backbone; it reflects a selection effect and identifies a critical evidence gap rather than a treatment effect. Adjuvant radiotherapy was rarely administered in the included studies and was not evaluated quantitatively.

Recent work in other gastrointestinal malignancies has suggested that systemic inflammatory and immune-nutritional indices may carry complementary prognostic information beyond pathological staging. The integration of such indices with TRG-based risk stratification has not been explored in the perioperative FLOT setting and represents a candidate direction for future multiparameter postoperative risk models [30,31].

Some cohorts have suggested differential postoperative benefit according to degree of regression [24,28], yet no prospective study has formally tested response-adapted postoperative strategies. Biomarker reporting remains limited. In one contemporary cohort, PD-L1 expression was observed in 53.4% of TRG2/3 patients (CPS 1–4: 20 cases; CPS 5–9: 12; CPS >10: 23), with no clear association to FLOT response; Claudin18.2 (26.2%), HER2 (7.8%), and MSI (3.9%) were infrequently expressed [24]. The heterogeneity and sparsity of molecular data preclude systematic inference, but underscore the need for integrated pathological and biomarker-driven evaluation in future trials.

Thirdly, sensitivity analyses and exploratory meta-regression did not identify major study-level covariates materially altering the pooled prognostic association. Although underpowered and interpreted cautiously, these analyses support the stability of the primary effect estimate across variations in covariate adjustment, resection status, and reporting practices. Risk of bias assessment further confirmed the retrospective nature of most cohorts and heterogeneity in adjustment strategies, reinforcing the need for standardized TRG-stratified reporting.

### 4.2. Clinical Implications

Pathological response assessment following perioperative FLOT provides an immediately available framework for postoperative risk stratification. Even after R0 resection, poor responders constitute a distinctly high risk phenotype and should be considered for intensified surveillance and clinical trial enrollment. At present, however, available data do not justify routine escalation, regimen switching, or radiotherapy incorporation outside prospective evaluation.

The emergence of perioperative chemoimmunotherapy intensifies this evidence gap. The recent *MATTERHORN* trial established FLOT plus durvalumab as a new standard [6], rapidly evolving the treatment landscape and reshaping expectations regarding residual disease biology. In this context, our meta-analysis establishes a rigorously quantified historical benchmark for the FLOT-only backbone. As immune-mediated mechanisms may alter traditional tumor regression patterns, defining the precise prognostic weight of residual disease under chemotherapy alone is essential for the accurate interpretation of emerging chemoimmunotherapy cohorts.

Future research priorities include the harmonization of TRG reporting across systems, standardized TRG-stratified postoperative outcome documentation, and prospective response adapted trials specifically targeting poor pathological responders. The integration of TRG with molecular and immune correlates and collaborative registry efforts will be essential to refine postoperative decision-making frameworks [19,20].

### 4.3. Limitations

Several limitations should be acknowledged. All included studies were observational and predominantly retrospective, introducing potential residual confounding despite multivariable adjustment, and they predate routine incorporation of perioperative immunotherapy, which may limit generalizability to evolving treatment paradigms. Although retrospective designs carry inherent selection bias, this is substantially mitigated by the robust statistical homogeneity of our primary overall survival analysis ($I^{2}=0.0\%$). Furthermore, our meta-regression confirmed that this adverse prognostic signal remains stable irrespective of baseline covariate adjustments or R0 resection status. Harmonization across Becker, Mandard, and CAP tumor regression grading systems may introduce misclassification; these frameworks use different histological thresholds and are not strictly interchangeable, meaning that the pooled estimate represents an average prognostic association across studies identifying limited regression rather than a risk estimate for a uniform biological phenotype. Nonetheless, subgroup analyses demonstrated consistent effect directionality across all three systems. Some cohorts applied broader definitions of poor response than others; Giommoni et al. classified all non-pCR patients as poor responders (prevalence 92.7%), while Biondi et al. restricted the category to Mandard TRG 5 (prevalence 16.7%), capturing materially different patient groups. Prognostic analyses were restricted to resected patients, reflecting postoperative clinical decision making but potentially introducing selection or survivor bias relative to intention-to-treat populations. For two studies, hazard ratios were reconstructed from reported summary statistics; exclusion of these cohorts did not materially alter pooled estimates. Finally, exploratory postoperative comparisons were based on three small non-randomized cohorts [13,28,29] and are susceptible to confounding by postoperative fitness; these findings should be interpreted as hypothesis-generating rather than evidence of therapeutic efficacy.

### 4.4. Interpretation in Context

Collectively, these findings establish poor pathological response after perioperative FLOT as a reproducible, high-impact prognostic marker while simultaneously defining a persistent therapeutic evidence gap. The prognostic signal remains stable across TRG systems, yet postoperative management strategies remain empirically undefined. As perioperative treatment paradigms evolve toward chemoimmunotherapy combinations, designing trials that incorporate TRG-defined risk stratification into postoperative decision making represents a clear priority for future research.

## 5. Conclusions

Poor pathological response after perioperative FLOT identifies a large and clinically meaningful high-risk subgroup among resected gastric and gastroesophageal junction adenocarcinoma patients, with approximately threefold inferior survival compared with responders. This meta-analysis establishes contemporary prognostic benchmarks across tumor regression grading systems and confirms the consistency of this adverse signal in the modern FLOT era.

At the same time, postoperative management for poor responders remains unsupported by prospective evidence. Although exploratory observational data suggest an association between completing planned postoperative therapy and improved outcomes, this comparison is non-randomized and inherently confounded by postoperative fitness and treatment selection; it should not be interpreted as evidence supporting the continuation of the same regimen in poor responders. The principal contribution of this study is therefore the quantification of both a robust prognostic effect and a major therapeutic evidence gap.

Prospective, response-adapted trials with harmonized pathological reporting and standardized postoperative outcome documentation are required to define optimal strategies for this high-risk population, particularly as perioperative treatment paradigms evolve toward chemoimmunotherapy combinations.

## Figures and Tables

**Table 1:** Study characteristics and extracted pathological response definitions.

Author (Year)	Country	Design	N Total	N Poor-Responders	Poor-Responder Rate	TRG System	Poor-Responder Definition	R0 (%)	No Surgery n (%)	Adj Given in Poor-Responders (%)	Median FU (mo.)
Al-Batran * 2019 [1]	Germany	Randomized controlled trial (RCT)	356	222	62.4%	Becker	TRG 2-3	85.0%	31 (9.0%)	60.0%	45
Giommoni 2021 [3]	Italy	Prospective cohort	190	176	92.7%	Becker	Non-pCR	92.1%	16 (7.8%)	74.7%	16
Erol 2022 [4]	Turkey	Retrospective cohort	441	315	76.2%	Becker	TRG 2-3	86.6%	28 (6.3%)	56.8%	13.5
Tomás 2022 [23]	Portugal	Retrospective cohort	295	193	33.5%	CAP	TRG 3	91.9%	0	NR	NR
Biondi 2023 [25]	Italy	Retrospective cohort	108	18	16.7%	Mandard	TRG 5	89.8%	0	NR	17.1
Möhring 2023 [5]	Germany	Retrospective cohort	55	32	58.2%	Becker	TRG 2-3	94.5%	0	85.5%	31.7
kee 2024 [26]	Singapore	Retrospective cohort	52	35	77.8%	CAP	TRG 2-3	88.5%	4	58.9%	16.9
Atci 2025 [27]	Turkey	Retrospective cohort	141	100	70.9%	CAP	TRG 2-3	94.8%	6	44.0%	12
Heckl 2025 [24]	Germany	Retrospective cohort	147	72	50.0%	Becker	TRG 3	90.3%	0	42.9%	NR
Kraemer 2025 [13]	Germany	Retrospective cohort	296	134	64.5%	Becker	TRG 2-3	100.0%	0	84.3%	28.2
Liu 2025 [28]	International	Retrospective cohort	1887	459	24.3%	3-tier (Complete, Partial, Minimal)	Grade 3	80.0%	0	59.3%	28.2
Sugiyama 2025 [29]	UK	Retrospective cohort	233	61	29.0%	Mandard	TRG 3–5	94.4%	0	78.9%	25.4

* For randomized trials, data refer only to the FLOT-treated arm.

**Table 2:** Survival outcomes and extracted effect estimates. For within-poor-pathological-responder comparisons, HR <1 favors completion of postoperative therapy.

Study	HR (OS): Non-Responders vs. Responders	HR (DFS/RFS): Non-Responders vs. Responders	HR (DFS) Adj vs. No-Adj in Non-Responders
Al-Batran, 2019 [1]	2.27 (1.47–3.57)	2.17 (1.45–3.23)	NR
Atci, 2024 [27]	33.10 (1.70–145.80)	32.80 (1.60–129.20)	NR
Biondi, 2023 [25]	4.87 (2.45–9.67)	5.17 (2.90–9.24)	NR
Erol, 2022 [4]	2.10 (1.06–4.15)	2.25 (1.09–4.65)	0.68 (0.47–0.98)
Giommoni, 2021 [3]	5.00 (1.23–20.00)	5.55 (1.35–25.00)	0.44 (0.22–0.86)
Heckl, 2025 [24]	NR	NR	NR
Kraemer, 2025 [13]	NR	NR	NR
Liu, 2025 [28]	NR	NR	NR
Möhring, 2023 [5]	2.80 (1.43–5.50)	2.81 (1.52–5.20)	NR
Sugiyama, 2025 [29]	2.21 (1.34–3.66)	2.05 (1.34–3.12)	0.31 (0.15–0.66)
Tomás, 2022 [23]	2.80 (1.60–5.00)	NR	NR
Kee, 2024 [26]	6.25 (0.70–50.00)	6.25 (0.70–50.00)	NR

**Table 3:** Subgroup meta-analysis by TRG system for OS.

TRG System	k	Pooled HR (95% CI)	I^2^ (%)	Tau^2^
Becker	4	2.44 (1.77–3.34)	0.0	0.000
CAP	3	6.11 (1.48–25.18)	57.7	0.925
Mandard	2	3.16 (1.46–6.84)	69.8	0.218

**Table 4:** Sensitivity analyses for prognostic OS.

Model	k	Pooled HR (95% CI)	I^2^ (%)	Tau^2^
Primary	9	2.73 (2.18–3.43)	0.0	0.000
Exclude Atci 2025	8	2.66 (2.12–3.34)	0.0	0.000
FU ≥ 24 months	4	2.43 (1.87–3.17)	0.0	0.000

**Table 5:** Meta-regression analyses of study-level moderators (OS).

Moderator	R^2^ (%)	Q_M	*p*-Value	Interpretation
Adjustment Status	0.0	0.62	0.430	Non-significant moderator
Non-Surgery Proportion	0.0	0.01	0.921	Non-significant moderator
R0 Resection Rate	0.0	0.42	0.515	Non-significant moderator

**Table 6:** Exploratory meta-analysis: completion of postoperative FLOT cycles versus no or incomplete postoperative therapy within poor pathological responders.

Analysis	k	Pooled HR (95% CI)	I^2^ (%)	Tau^2^
Exploratory pooled (adjuvant vs. no adjuvant)	3	0.49 (0.31–0.79)	50.1	0.089

**Table 7:** Adjusted covariates in reported hazard ratio models.

Study	Adjusted Covariates
Al-Batran, 2019 [1]	Age, sex, Lauren, location, ypTN (in later subgroup analyses)
Möhring, 2023 [5]	Age, Sex, pT, pN, TRG, Adjuvant chemo completion
Atci, 2024 [27]	Univariable only
Kraemer, 2025 [13]	Age, sex, tumor localisation, histology, signet ring cells, ypT, ypN, surgical approach
Kee, 2024 [26]	Univariate only
Sugiyama, 2025 [29]	Not reported
Tomas, 2022 [23]	T-stage regression, NLR, TRG
Liu, 2025 [28]	Propensity score matching
Erol, 2022 [4]	NLR
Heckl, 2025 [24]	Not reported
Biondi, 2023 [25]	Charlson Index, HER2
Giommoni, 2021 [3]	Not reported

## Data Availability

Data were extracted from publicly available sources. The dataset is available from the corresponding author upon request.

## Supplementary Materials

The following supporting information can be downloaded at https://www.mdpi.com/article/10.3390/jcm15062367/s1: Figure S1: Full database search strategies (Web of Science, Scopus, Cochrane Library); Figure S2: Study-level forest plots by TRG subgroup (OS); Figure S3: Study-level forest plots by TRG subgroup (DFS/RFS); Figure S4: Leave-one-out sensitivity analysis plots; Figure S5: Funnel plot for DFS/RFS publication bias assessment; Figure S6: Descriptive distribution of post-treatment pathological T-stage (ypT) among poor responders; Figure S7: Descriptive distribution of post-treatment nodal status (ypN) among poor responders; Table S1: Newcastle–Ottawa Scale risk of bias assessment; Table S2: Becker TRG subgroup meta-analysis (OS and DFS/RFS); Table S3: Mandard TRG subgroup meta-analysis (OS and DFS/RFS); Table S4: CAP TRG subgroup meta-analysis (OS and DFS/RFS); Table S5: Meta-regression results (DFS/RFS); Table S6: Leave-one-out sensitivity analysis results; Table S7: Molecular biomarker reporting summary across included studies.

## Abbreviations

The following abbreviations are used in this manuscript:

CAP	College of American Pathologists
CI	Confidence Interval
CPS	Combined Positive Score
DFS	Disease-Free Survival
FLOT	Fluorouracil, Leucovorin, Oxaliplatin, and Docetaxel
GEJ	Gastroesophageal Junction
HER2	Human Epidermal Growth Factor Receptor 2
HR	Hazard Ratio
MSI	Microsatellite Instability
NOS	Newcastle–Ottawa Scale
OS	Overall Survival
PD-L1	Programmed Death-Ligand 1
PFS	Progression-Free Survival
PICO	Population, Intervention, Comparison, Outcome
PRISMA	Preferred Reporting Items for Systematic Reviews and Meta-Analyses
PROSPERO	International Prospective Register of Systematic Reviews
REML	Restricted Maximum Likelihood
RFS	Recurrence-Free Survival
TRG	Tumor Regression Grade
