# Supplementary material for: Prognostic Impact and Postoperative Management Following Poor Pathological Response to Perioperative FLOT in Resectable Gastric and GEJ Adenocarcinoma: A Systematic Review and Meta-Analysis
Source: J Clin Med. 2026 Mar 20;15(6):2367. doi: 10.3390/jcm15062367 (PMC13026895; doi:10.3390/jcm15062367)
Supplement: Supplementary file 1 [file jcm-15-02367-s001.zip › jcm-4187253-supplementary.pdf]

Supplementary Appendix

Prognostic Impact and Postoperative Management Following Poor Pathological Response to Perioperative FLOT in Resectable Gastric and GEJ Adenocarcinoma: A Systematic Review and Meta-analysis

Guide to Supplementary Materials

This appendix contains the full electronic search strategies, additional descriptive and technical analyses, the PRISMA 2020 checklist, and the complete list of excluded full-text studies with reasons.

Supplementary Methods

Full electronic search strategies

PubMed/MEDLINE

("gastric cancer"[Title/Abstract] OR "gastroesophageal junction cancer"[Title/Abstract] OR "stomach neoplasms"[MeSH Terms] OR "gastric adenocarcinoma"[Title/Abstract] OR "GEJ cancer"[Title/Abstract] OR "oesophagogastric cancer"[Title/Abstract]) AND ("FLOT"[Title/Abstract] OR ("docetaxel"[Title/Abstract] OR "oxaliplatin"[Title/Abstract] OR "leucovorin"[Title/Abstract] OR "5-fluorouracil"[Title/Abstract]) OR "perioperative chemotherapy"[Title/Abstract] OR "neoadjuvant chemotherapy"[Title/Abstract] OR "adjuvant chemotherapy"[Title/Abstract] OR "triplet chemotherapy"[Title/Abstract]) AND ("tumor regression grade"[All Fields] OR "TRG"[All Fields] OR "pathologic response"[All Fields] OR "histopathologic response"[All Fields] OR "responder"[All Fields] OR "non-responder"[All Fields] OR "partial response"[All Fields] OR "pCR"[All Fields] OR "treatment response"[All Fields]) AND ("R0 resection"[All Fields] OR "curative surgery"[All Fields] OR "radical surgery"[All Fields] OR "overall survival"[All Fields] OR "OS"[All Fields] OR "progression free survival"[All Fields] OR "PFS"[All Fields] OR "disease free survival"[All Fields])

Web of Science Core Collection

TS=("gastric cancer" OR "gastroesophageal junction cancer" OR "stomach neoplasms" OR "gastric adenocarcinoma" OR "GEJ cancer" OR "oesophagogastric cancer") AND TS=("FLOT" OR "docetaxel" OR "oxaliplatin" OR "leucovorin" OR "5-fluorouracil" OR "perioperative chemotherapy" OR "neoadjuvant chemotherapy" OR "adjuvant chemotherapy" OR "triplet chemotherapy") AND TS=("tumor regression grade" OR "TRG" OR "pathologic response" OR "histopathologic response" OR "responder" OR "non-responder" OR "partial response" OR "pCR" OR "treatment response") AND TS=("R0 resection" OR "curative surgery" OR "radical surgery" OR "overall survival" OR "OS" OR "progression free survival" OR "PFS" OR "disease free survival")

Scopus

TITLE("gastric cancer" OR "gastroesophageal junction cancer" OR "gastric adenocarcinoma") AND TITLE-ABS-KEY("FLOT" OR "docetaxel" OR "oxaliplatin" OR "triplet chemotherapy") AND TITLE-ABS-KEY("tumor regression grade" OR "TRG" OR "pathologic response" OR "pCR") AND TITLE-ABS-KEY("R0 resection" OR "overall survival" OR "PFS" OR "disease free survival")

Cochrane Library

("gastric cancer" OR "gastric adenocarcinoma" OR "gastroesophageal junction cancer") AND (FLOT OR "neoadjuvant chemotherapy" OR "adjuvant chemotherapy") AND ("tumor regression grade" OR TRG OR "pathologic response") AND ("overall survival" OR OS OR "progression free survival" OR PFS)

Supplementary Results

Supplementary Table S1. Risk of bias assessment (Newcastle–Ottawa Scale)

Table S1: Risk of bias assessment of included observational cohort studies using the Newcastle–Ottawa Scale (NOS). Selection (S1–S4; 0/1 each), Comparability (C; 0–2), Outcome (O1–O3; 0/1 each). Maximum score 9.

Study	S1	S2	S3	S4	C	O1	O2	O3	Total
Giommoni 2021	1	1	1	1	2	1	1	1	9
Erol 2022	1	1	1	1	1	1	1	1	8
Tomas 2022	1	1	1	1	1	1	0	0	6
Biondi 2023	1	1	1	1	1	1	0	0	6
Mohring 2023	1	1	1	1	1	1	1	0	7
kee 2024	1	1	1	1	1	1	0	0	6
Atci 2025	1	1	1	1	1	1	1	1	8
Heckl 2025	1	1	1	1	1	1	0	0	6
Kraemer 2025	1	1	1	1	2	1	1	1	9
Liu 2025	1	1	1	1	2	1	1	0	8
Sugiyama 2025	1	1	1	1	2	1	1	1	9

Legend: S1 representativeness; S2 same-source comparison cohort; S3 ascertainment of exposure; S4 outcome not present at baseline; C adjustment (0–2); O1 outcome assessment; O2 follow-up duration; O3 adequacy of follow-up.

Supplementary Figure S1. Baseline characteristics forest plot

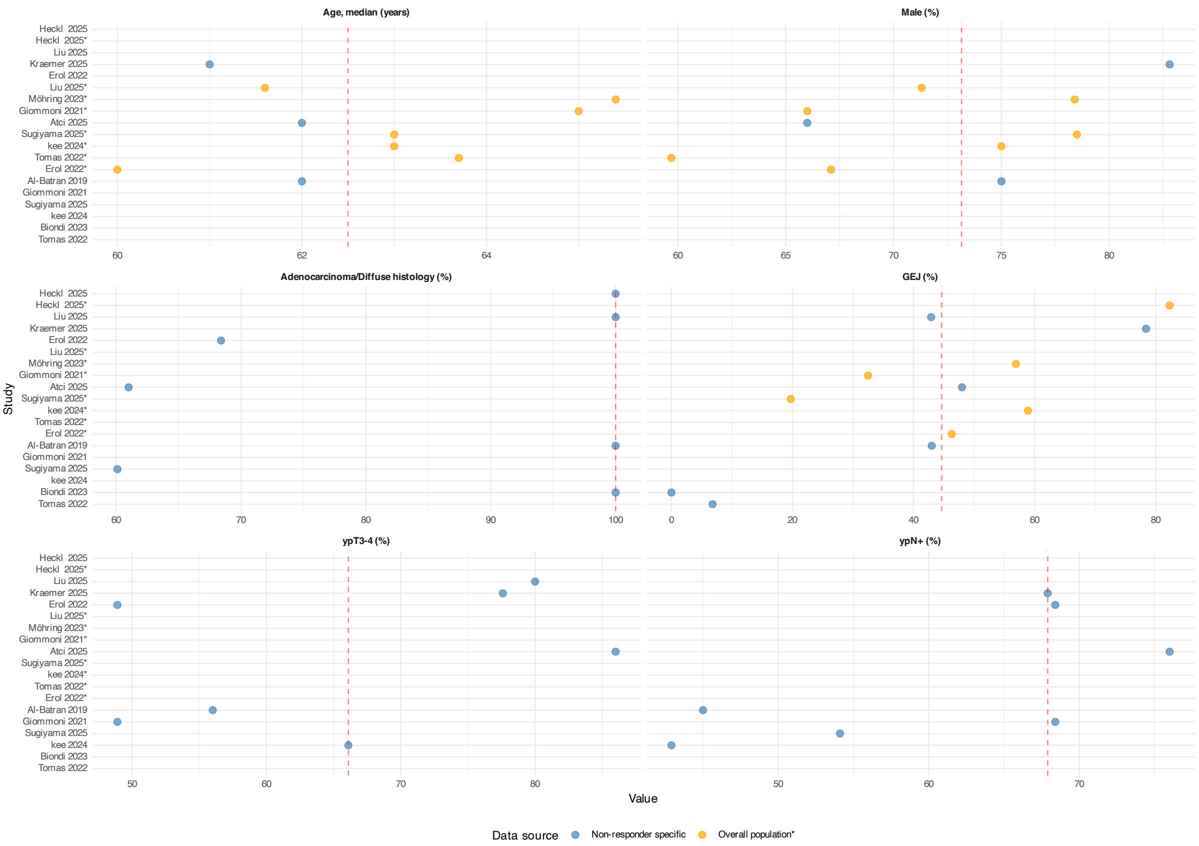

Figure S1: Baseline characteristics of pathological poor responders (forest plot). Values marked with * are derived from overall cohorts rather than poor responder–specific reporting.

Supplementary Table S2. Baseline characteristics of poor pathological responders

Table S2: Baseline characteristics of poor pathological responders.

Study	N non-responders	Age, median (years)	Male (%)	Adenocarcinoma/Diffuse histology (%)	Intestinal histology (%)	GEJ (%)	ypT3-4 (%)	ypN+ (%)
Al-Batran 2019	222	62.0	75.0%	100.0%	NR	43.0%	56.0%	45.0%
Atci 2025	100	62.0	66.0%	61.0%	NR	48.0%	86.0%	76.0%
Biondi 2023	18	NR	NR	100.0%	NR	0.0%	NR	NR
Erol 2022	315	60.0*	67.1%*	68.4%	NR	46.3%*	48.9%	68.4%
Giommoni 2021	176	65.0*	66.0%*	NR	NR	32.5%*	48.9%	68.4%
Heckl 2025	72	NR	NR	100.0%	NR	82.3%*	NR	NR
Kraemer 2025	134	61.0	82.8%	NR	48.5%	78.4%	77.6%	67.9%
Liu 2025	459	61.6*	71.3%*	100.0%	NR	42.9%	80.0%	NR
Möhring 2023	32	65.4*	78.4%*	NR	71.7%	56.9%*	NR	NR
Sugiyama 2025	61	63.0*	78.5%*	60.1%	NR	19.7%*	NR	54.1%
Tomas 2022	193	63.7*	59.7%*	NR	NR	6.8%	NR	NR
kee 2024	35	63.0*	75.0%*	NR	NR	58.9%*	66.1%	42.9%

Values marked with * are derived from overall cohorts rather than poor responder–specific reporting.

Supplementary Figure S2. R0 resection rate

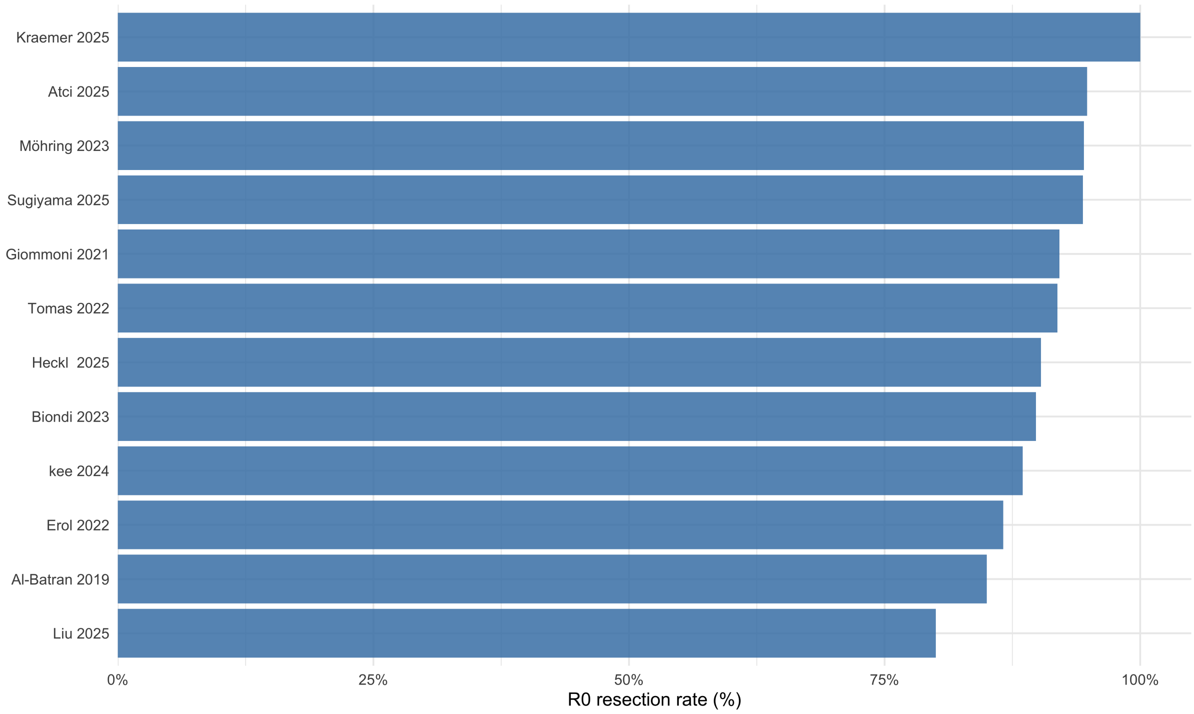

Figure S2: R0 resection rate across studies.

Supplementary Figure S3. Publication bias assessment (DFS/RFS)

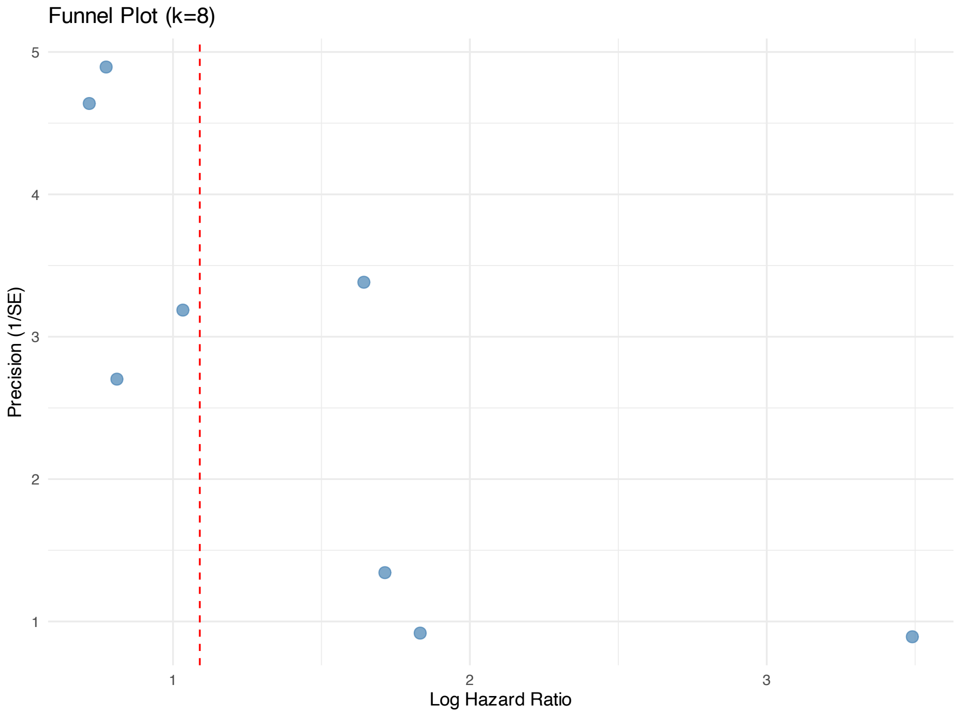

Figure S3: Funnel plot for assessment of publication bias (DFS/RFS). Egger regression test $p > 0.05$.

Supplementary Figure S4. Leave-one-out analysis (OS)

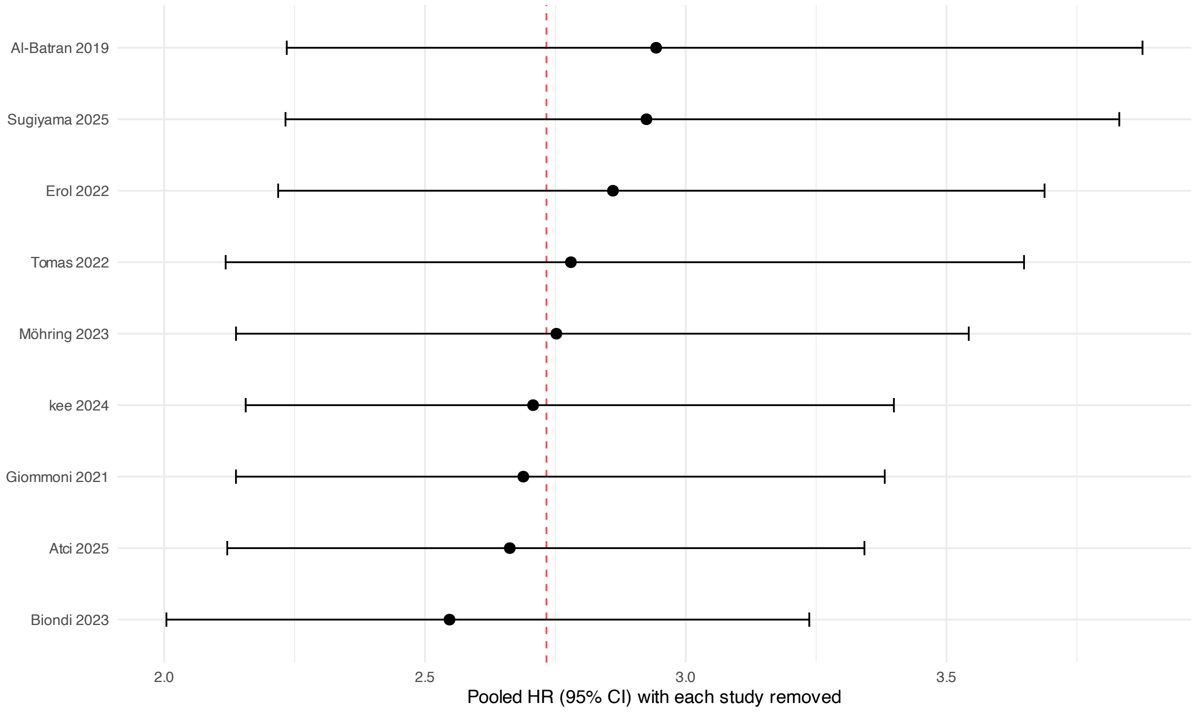

Figure S4: Leave-one-out analysis for prognostic OS (poor responders versus responders).

Supplementary Figure S5. Leave-one-out analysis (postoperative comparison)

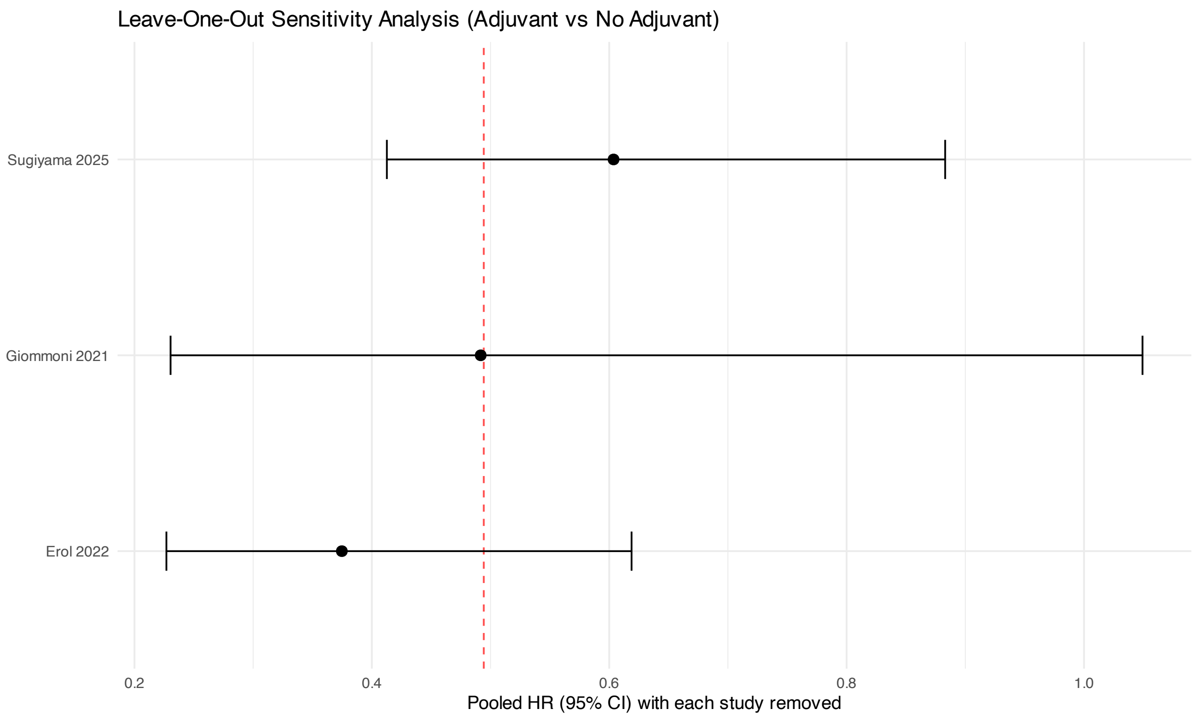

Figure S5: Leave-one-out analysis for completion of postoperative FLOT cycles versus no or incomplete therapy within poor responders (exploratory).

Supplementary Table S3. Exploratory meta-analysis: completion of postoperative FLOT cycles versus no or incomplete therapy

Table S3: Exploratory meta-analysis: completion of postoperative FLOT cycles versus no or incomplete postoperative therapy within poor responders.

Analysis	k	Pooled HR (95% CI)	I^2 (%)	Tau^2
Exploratory pooled (adjuvant vs no adjuvant)	3	0.49 (0.31–0.79)	50.1	0.089

Supplementary Table S4. Adjusted covariates in reported hazard ratio models

Table S4: Adjusted covariates in reported hazard ratio models.

Study	Adjusted covariates
Al-Batran 2019	Age, sex, Lauren, location, ypTN (in later subgroup analyses)
Möhring 2023	Age, Sex, pT, pN, TRG, Adjuvant chemo completion
Atci 2025	Univariable only
Kraemer 2025	Age, sex, tumour localisation, histology, signet ring cells, ypT, ypN, surgical approach
kee 2024	Univariate only
Sugiyama 2025	Not reported
Tomas 2022	T-stage regression, NLR, TRG
Liu 2025	Propensity score matching
Erol 2022	NLR
Heckl 2025	Not reported
Biondi 2023	Charlson Index, HER2
Giommoni 2021	Not reported

Supplementary Figure S6. Tumor biology characteristics among pathological poor responders

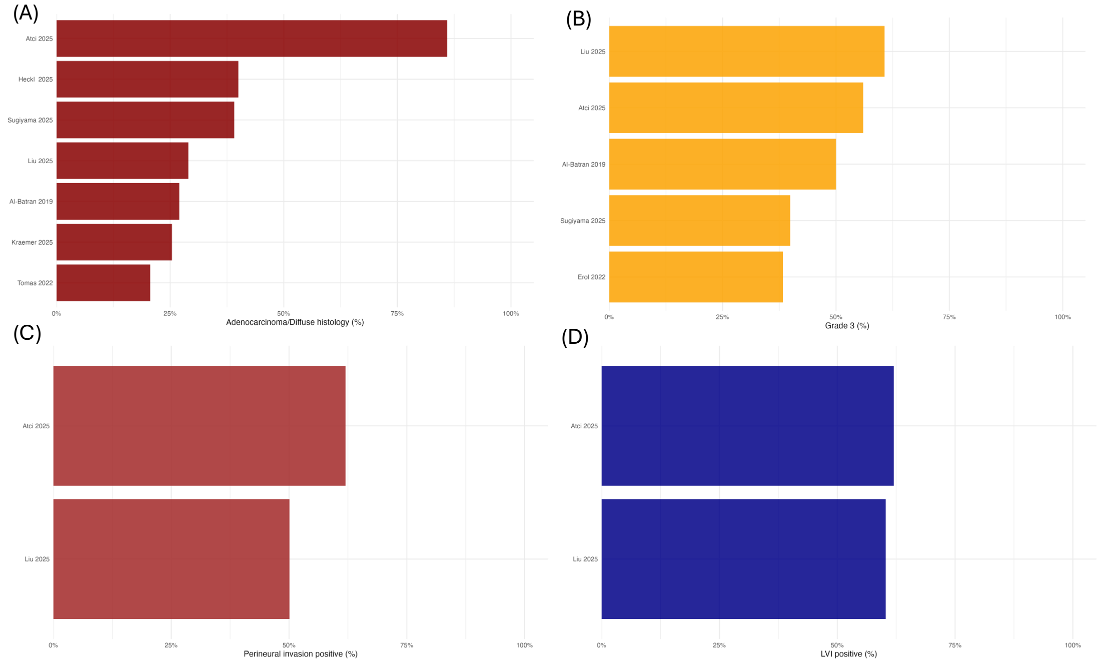

Figure S6: (A) Lauren classification; (B) Tumor grade distribution; (C) Perineural invasion;(D) Lymphovascular invasion among pathological poor responders.

Supplementary Figure S7. Post-treatment staging and recurrence patterns

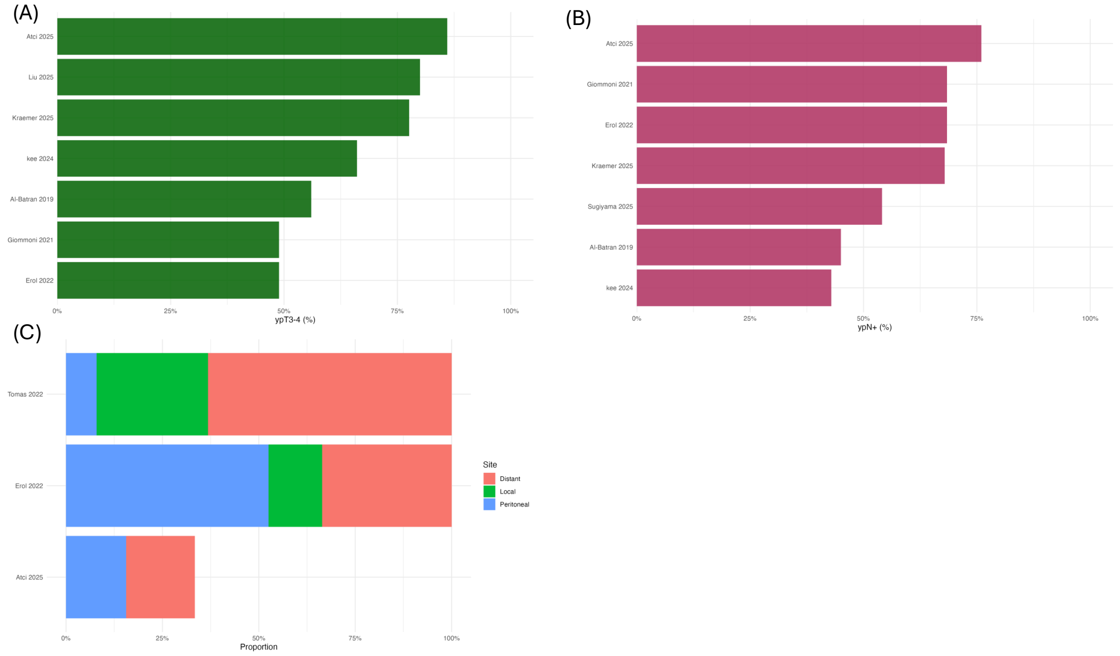

Figure S7: (A) Pathological T stage (ypT3–4); (B) Pathological N stage (ypN+); (C) Recurrence pattern distribution among poor responders.

Supplementary Table S5. PRISMA 2020 Checklist

Section	Item	Checklist Item	Location in Manuscript
Title	1	Identify the report as a systematic review.	Title page
Abstract	2	See PRISMA 2020 for Abstracts checklist.	Structured abstract
Introduction	3	Describe rationale.	Introduction
	4	Explicit objectives.	Final paragraph of Introduction
Methods	5	Eligibility criteria.	Methods: Eligibility, selection, extraction and synthesis
	6	Information sources.	Methods: Information sources and search strategy
	7	Full search strategies.	Methods (PubMed string) + Supplementary Appendix
	8	Selection process.	Methods: Eligibility, selection, extraction and synthesis
	9	Data collection process.	Methods: Eligibility, selection, extraction and synthesis
	10a	Outcomes sought.	Methods: Eligibility, selection, extraction and synthesis
	10b	Other variables sought.	Methods: Eligibility, selection, extraction and synthesis
	11	Risk of bias assessment.	Methods: Eligibility, selection, extraction and synthesis
	12	Effect measures.	Methods: Eligibility, selection, extraction and synthesis
	13a–f	Synthesis methods.	Methods: Eligibility, selection, extraction and synthesis
	14	Reporting bias assessment.	Methods: Eligibility, selection, extraction and synthesis
	15	Certainty assessment.	Not performed
Results	16a	Study selection flow.	Figure 1 (PRISMA diagram)
	16b	Excluded studies explanation.	Results: Study selection + Supplementary Table S6
	17	Study characteristics.	Table 1
	18	Risk of bias results.	Table 2
	19	Individual study results.	Table 3 + Forest plots
	20a–d	Results of syntheses.	Results: Oncologic outcomes
	21	Reporting bias results.	Supplementary Figures S3 and S5
	22	Certainty of evidence.	Not performed
Discussion	23a–d	Interpretation, limitations, implications.	Discussion section

Other	24a	Registration.	Methods: Reporting standard and protocol
	25	Support.	Funding statement
	26	Competing interests.	Conflicts of Interest
	27	Data availability.	Data Availability statement

Supplementary Table S6. Excluded full-text studies with reasons

Study ID	PRISMA Category	Reason for Exclusion (Short)
Sajjadi_2024	No TRG Stratification	Descriptive analysis of pathologic response rates only; no survival outcomes reported.
Egebjerg_2024	No TRG Stratification	Reports completion rates and pCR only; no survival data stratified by response.
López_2024	No TRG Stratification	Outcomes aggregated for the entire FLOT arm; no responder vs. non-responder survival analysis.
Glatz_2015	No TRG Stratification	Single-arm feasibility/safety study; reports aggregate survival but no stratification by TRG.
Serra_2025	No TRG Stratification	Descriptive cohort study; lacks survival curves specifically for the non-responder subgroup.
Sisic_2023	No TRG Stratification	Focuses on surgical outcomes (morbidity/mortality); no oncologic survival by response grade.
Sinnamon_2023	No TRG Stratification	Analyzes predictors of pCR; does not report longitudinal survival outcomes for failures.
Brunner_2024	Study Design	Conference abstract only; no full-text publication with extractable data.
Gan_2025	No TRG Stratification	Reports pCR rates as primary endpoint; survival data not stratified.
Gal_2024	No TRG Stratification	Reports pathological results; no Kaplan–Meier curves for non-responders.
Spoerl_2018	No TRG Stratification	Focuses on immunological predictors of response; survival not stratified by TRG.
Li_2025	No TRG Stratification	Descriptive retrospective series; aggregate outcomes only.
Cai_2020	No TRG Stratification	Safety and feasibility focus; no survival breakdown by pathological response.
Abboretti_2024	No TRG Stratification	Reports pCR rates; lack of stratified OS/DFS data for non-responders.
Zhong_2025	No TRG Stratification	Focuses on pathological response; survival data not provided.
Ding_2024	Ineligible Intervention	Mixed cohort (XELOX + SOX); no data for FLOT.
Geerts_2024	No TRG Stratification	Real-world data on pCR; no survival outcomes for non-pCR group.
Tian_2024	No TRG Stratification	Reports pCR/downstaging only; no survival stratification.
Sah_2020	No TRG Stratification	Safety and feasibility focus; no survival outcomes reported.
Kumar_Sah_2025	No TRG Stratification	Secondary outcomes/safety; no specific non-responder survival curves.
Kumar_Sah_2021	No TRG Stratification	Feasibility/Safety pilot; no long-term survival data.
Al-Batran_2016	No TRG Stratification	Phase 2 endpoint (pCR) only; survival data was immature (superseded by later report).
Goetze_2023	No TRG Stratification	Primary endpoint was R0/pCR; survival not stratified by TRG in this publication.

Lorenzen_2024	No TRG Stratification	Interim analysis of pCR/safety; survival data immature/not reported.
Hofheinz_2022	No TRG Stratification	Small sample size (terminated early); no meaningful survival stratification by response.
Schulz_2015	No TRG Stratification	Single-arm Phase II; reports aggregate DFS/OS but no curve for non-responders.
Hoeppner_2025	No TRG Stratification	Primary comparison FLOT vs. CROSS; survival not stratified by TRG within FLOT arm.
Shitara_2024	No TRG Stratification	Primary comparison pembrolizumab vs. placebo; survival not stratified by TRG in the control arm.
Leong_2024	Ineligible Intervention	Mixed cohort (FLOT + ECF/ECX); data for FLOT non-responders cannot be isolated.
Zaidi_2021	Ineligible Intervention	Mixed cohort (perioperative chemotherapy vs. postoperative chemoradiotherapy); intervention not strictly perioperative FLOT.
Wagner_2019	Study Design	Study protocol document only; contains no results.

Table S7: Supplementary Table S7. Reporting of molecular biomarkers across included studies.

Study	HER2	PD-L1	MSI/MMR	CLDN18.2	Other biomarkers	Stratified by TRG
Al-Batran 2019 (FLOT4)	NR	NR	NR	NR	None reported	No
Moehring 2023	NR	NR	NR	NR	None reported	No
Atci 2025	No	No	No	No	CEA, CA19-9	Yes
Kraemer 2025	Yes	Yes	Yes	No	–	No
Kee 2024	Yes	No	Yes	No	–	No
Sugiyama 2025	Yes	No	Yes	No	–	No
Tomas 2022	NR	NR	NR	NR	None reported	No
SPACE-FLOT 2025	NR	NR	NR	NR	None reported	No
Erol 2022	Yes	No	No	No	–	No
Heckl 2025	Yes	Yes	Yes	Yes	–	Yes
Biondi 2023	Yes	No	No	No	Albumin	Yes
Giommoni 2021	No	No	Yes	No	–	No

NR = not reported. MMR = mismatch repair. MSI = microsatellite instability. CLDN18.2 = Claudin-18.2. Biomarker reporting across the included studies was inconsistent and rarely stratified by pathological response category.
